# Retraction Note: Titanium particles damage osteocytes and inhibit osteoblast differentiation

**DOI:** 10.1186/s40634-020-00304-z

**Published:** 2020-11-03

**Authors:** Li Chen, Ziyue Wang, Wei Xu, Qirong Dong

**Affiliations:** 1 Second Department of Orthopaedics, SuZhou Municipal Hospital, Suzhou City, Anhui Province China; 2 Department of Orthopedics, The Second Affiliated Hospital of Soochow University, Suzhou City, Jiangsu Province China

**Correction to: Journal of Experimental Orthopaedics 7, 47 (2020)**

10.1186/s40634-020-00268-0

The authors have retracted this article [1] because after publication it became apparent that there were problems with the method used for the cultivation of the osteocytes. The data presented are therefore unreliable. All authors agree with this retraction.
